# A citizen-based assessment of European smart cites using a DP2 index

**DOI:** 10.1038/s41598-026-55659-7

**Published:** 2026-05-30

**Authors:** Tiziano Pavanini, Andrea Ciacci, Tommaso Filì, Enrico Ivaldi

**Affiliations:** 1 Department of Architecture and Urban Studies (DAStU), Politecnico di Milano, Milan, Italy; 2 Department of Economics, University of Genoa, Genova, Italy; 3 Italian Centre of Excellence in Logistics, Transport and Infrastructure (CIELI), University of Genoa, Genova, Italy; 4 Department of Humanistic Studies, IULM University - Faculty of Communication, Milan, Italy

**Keywords:** Smart cities, Citizen perceptions, DP2 method, Urban sustainability, Digital–infrastructure integration, Development studies, Environmental social sciences, Geography, Geography, Science, technology and society

## Abstract

Despite the growing body of research on smart cities, existing studies tend to rely on objective indicators, often overlooking how citizens perceive the balance between key urban dimensions. To fill this gap, the present research analyzes the current development of 56 smart cities in Europe through a multidimensional assessment framework based on the non-compensatory Distance-Pena (DP2) method. While most assessments of smart cities use objective measures to gauge their performance, this research emphasizes citizens’ viewpoints by assessing perceptions concerning the infrastructural and technological dimensions of smart cities within five categories, namely health and safety, mobility, activities, opportunities, and governance. The results identify four different types of cities based on their respective levels of structural and technological performances. In some cases, there is equal development on both fronts, while in others, there is unequal development, characterized by a strong presence in either one aspect or another. These patterns generate heterogeneous configurations of perceived smart city performance. The findings provide a descriptive typology of smart cities based on citizen perceptions and offer insights into how different dimensions of urban performance can be evaluated. This study adds to the conceptual knowledge of smart cities and provides practical value for policymakers designing smart city strategies that align with citizen-centric views.

## Introduction

Smart cities (SCs) represent an innovative urban model that integrates advanced technologies with the objective of improving the quality of life of citizens, promoting environmental sustainability and fostering economic growth^1^. However, as the development of smart cities is not linear and uniform^2–4^, analyzing cross-sectional differences in their relative perceived performance can help identify variations in how cities perform across key dimensions and inform policy-relevant comparisons. This is beneficial to policymakers and planners, as it can lead to more effective strategies to promote urban development.

Academic research on SCs, in fact, necessitates a systemic and comparative approach, capable of highlighting the differences and similarities between the various urban realities^2^ and conceptual constructs^5^. Considering this, the present study aims to provide a comparative and cross-sectional assessment of smart cities in Europe, with a view to classifying cities into four groups based on their relative performance, and to provide an interpretative framework of European cities grounded in citizens’ perceptions of structural and technological dimensions.

Adopting a citizen-centric perspective, as frequently overlooked in scientific research^6^ yet emphasized by Chang et al.,^7^, we integrate the human aspect with the structural and technological considerations associated with the development of smart cities. This approach enables the integration of the two fundamental pillars of smart cities, namely technology-driven and human-driven methods^5^.

This study thus seeks to address the gap in existing literature by providing a comparative analysis of citizen perceptions across 56 European cities, focusing on the infrastructural (“*structures*”) and technological dimensions of smart city performance^8,9^. To this end, data from the Smart City Index report was extracted and analyzed in accordance with the methodology employed by the abovementioned authors in their own study.

Since traditional indicators employed may be limited in capturing intricate phenomena, such as the level of smartness and sustainability exhibited by cities, resulting in the obfuscation of critical issues in specific dimensions, the generation of information redundancy, and the introduction of distortions, in this study we elected to utilize the Distance-Pena (DP2) method, a non-compensatory technique for the construction of aggregate indices. A review of the literature indicates that the application of the DP2 method in the context of smart cities remains underexplored, although contributions such as Ciacci et al.,^10^ illustrate its potential for constructing composite indices based on territorial data. In their future agenda, the authors emphasize the necessity for an approach that is more aligned with the impressions of citizens and, consequently, data that are derived from their perceptions.

The present study contributes to this discourse by adopting a perception-based, non-compensatory comparative framework. It focuses on the classification and interpretation of cities according to citizen-perceived structural and technological performance, without seeking to infer transition mechanisms or temporal dynamics.

This paper is comprised of five sections. The first, entitled “Introduction,” presents the research questions and the aim of this work. Second, a literature review analyses the state of the art of academic literature on SCs, highlighting leading cities in different categories. The third section, entitled “Methodology,” presents the data and the applied statistical method. The results are stated in the fourth section, entitled “Results,” and discussed in the fifth section, “Discussion.” The final section, labelled “Conclusion,” exposes the limitations of this study and the future agenda.

## Literature review

To date, a plethora of studies of the development of smart cities have been conducted in academic literature. These analyses have employed a variety of distinct yet, in some cases, similar methodologies and approaches. A substantial number of analysis concentrate on the formulation of specific indicators for the measurement and classification of SCs’ performances: for instance, the Slovak Smart City Index developed by Kona et al.,^11^ and an evaluation index of Chinese SCs^12^. Furthermore, a few studies have investigated the clustering and ranking techniques employed in the analysis of SCs (e.g.^8,13^.

Much of the current literature on smart city leverages mainly objective indicators, with less attention devoted to perception-based assessments of smart city performance^14^. In this regard, the study by Toh^9^ is significant as it shows that out of 6 of the main indices developed, 5 use objective data and only one, the Smart City Index (the same one we refer to in this paper) uses perception-based data. This point is relevant because subjective evaluations are capable of identifying some aspects of life in an urban environment that cannot be identified through objective measures. Moreover, existing techniques rely on compensatory aggregation, which may limit the role of individual factors and allow for compensation where high scores in one area can make up for low scores in another area. These limitations are what encourage the use of non-compensatory techniques, such as DP2.

The following subsections present the main directions of academic literature on SCs, with a focus on how structural and technological dimensions are conceptualized and measured. The reviewed studies are grouped into three categories: SCs as multi-infrastructural systems (2.1), SCs as techno-centric entities (2.2) and SCs that integrate both dimensions in their identity (2.3).

### Smart cities as multi-infrastructural systems

Infrastructure plays a vital role in the development of smart cities, providing a foundation for the integration of smart technologies that enhance the quality of life for citizens, enhance urban efficiency, and promote environmental sustainability. This literature direction views physical and organizational infrastructures as key components of urban performance. SCs depend on the integration of information and communication technologies (ICT) with existing infrastructure, necessitating investments in sectors such as energy, water, transport, waste management and telecommunications^15–17^. The development of smart infrastructure is a crucial step in the effort to enhance climate resilience.

### Smart cities as techno-centric entities

Several enabling technologies, including edge computing, unmanned aerial vehicles, and blockchain, play a pivotal role in the development and sustainability of smart cities (Khan et al., 2019)^18,19^. For instance, edge computing has been identified as a key component in the realization of the vision of smart cities, optimizing resource utilization and enhancing operations in various sectors, including health, transportation, energy, and water services (Khan et al., 2019).

As the concept of smart cities continues to evolve, there is a growing emphasis on the importance of technical standards to ensure interoperability and seamless integration of various technologies^20^. Additionally, the concept of digital twins emerges as a key technology for smart cities, offering opportunities for advanced simulations and monitoring of urban environments^21^. The literature reveals a multitude of technologies and methodologies being investigated with the aim of creating sustainable and efficient smart cities. These endeavors are accompanied by an examination of pertinent issues such as cybersecurity, interoperability, and energy conservation^22,23,64^.

### The integration of structural and technological dimensions in the smart city’s identity

The concept of SCs has received considerable attention in recent years, with a focus on integrating information and communication technology (ICT) with traditional city infrastructure to enhance efficiency and services for residents^24^.

In this scenario, the governance and information systems play a pivotal role in the evolution of smart cities: Zhou et al.,^25^ conducted a scientometric analysis to investigate the trends in SCs research, while Gupta^26^ stated that to achieve human-centric SCs, it is essential that policy makers can measure and monitor the environmental and social impacts over time.

The integration of structural and technological dimensions in SCs presents several challenges that must be addressed to ensure its successful implementation such as the issue of security within SC networks, the privacy of individuals and data protection^62,66,67^.

The following subsections (2.3.1, 2.3.2, 2.3.3 and 2.3.4) present the leading European cities for each of the city group identified in this study.

#### Integrated innovation pioneers

This subsection enumerates some of Europe’s most prominent cities in terms of their holistic and ecosystemic integration. For example, **Zurich** excels in structures and technologies, showing advanced public transportation systems, renewable energy adoption, and digital governance platforms. Smart City Zurich Strategy prioritizes synergy between technology and sustainable urban development, with initiatives such as autonomous public transport and advanced smart grid systems^27,28^.

**Copenhagen** has set itself the ambitious goal of becoming carbon-neutral by 2025 and is frequently regarded as one of the most advanced cities in terms of integrating all dimensions of sustainability. The Danish capital offers its residents a relatively high quality of life and well-being. Moreover, the city demonstrates a notable proficiency in the domain of efficient urban transportation. Furthermore, citizens have access to over 400 km of cycle paths, with 62% of these being utilised on a daily basis^29,30^. Furthermore, Copenhagen has enhanced its digital infrastructure, thereby facilitating convenient access for citizens to the capital’s online services.

#### Techno-centric innovators

Techno-centric cities have prioritised the development of their digital grid, with a concomitant neglect of investment in physical infrastructure. This has resulted in an incomplete integration of the two dimensions. **Warsaw** has leveraged digital innovation, with projects such as the Virtual Warsaw app, a beacon-based navigation system for visually impaired citizens, and e-services for administrative procedures, to achieve higher relative perceived performance. The city’s strategy reflects a push for digital transformation in public services^31^.

**London** stands out for the Smarter London Together project launched by the mayor of the city in 2018, which aims to digitally transform the British capital and make it one of the smartest cities in the world. London has invested in citizen-friendly services and big data management, focusing its efforts mainly on governance and innovation^32–34^. The city now needs to better integrate the digital aspect with the physical infrastructure it already has.

#### Transitioning cities

Cities undergoing a transition are demonstrating the least progress in achieving sustainability. These cities are experiencing difficulties in both infrastructural and digital development. **Bucharest** faces infrastructural challenges, such as aging public transport systems and outdated infrastructure^35^, compounded by slow digital adoption. While the city has launched some development strategies (e.g., the deployment of smart meters for energy management)^36^, these initiatives may lack ecosystemic integration into a broader urban development framework in the early stages of implementation.

**Rome** is often cited among cities with difficulties in digital transformation. Despite the approval of the Roma Smart City project approved in March 2021^37^, the Italian capital shows a significant delay in the implementation of smart technologies. Problems in managing public services and obsolete infrastructure hinder progress towards greater efficiency and sustainability.

#### Resilient cities

The concept of a resilient city places greater emphasis on the infrastructural dimension, with less attention paid to the digital aspect. **Hamburg** shows high-quality physical infrastructures, including advanced logistics systems, urban planning excellence, and environmental sustainability projects such as the HafenCity redevelopment^38–40^.

**The Hague** has adopted a strategy based on the development of a robust physical infrastructure as a foundation for its smart city transition. This strategy encompasses investments in sustainable mobility^41^, urban renewal^42^ and energy network modernization^43^. These endeavors are designed to enhance the quality of life and advance sustainability. While digital initiatives, such as smart grids and monitoring systems, are present, they are less developed than in other European cities and still in their infancy. This is due to the predominantly public-sector and less technology-based economy of the city^44^.

## Methods

### Data

The analysis data comes from the Smart City Report developed in 2023 by the IMD International Institute for Management Development at the Singapore University of Technology and Design. This report aims to enable the benchmarking of cities at a global scale and establish their development level. The report collects data on smart cities based on people’s perceptions through specific surveys^45^. This approach aims to understand the needs of residents, providing policy makers with important information on subjective assessments of urban contexts.

The report collects data on 141 cities across the world. However, to facilitate data comparability and reduce any bias, we decided to focus the analysis on a more homogeneous territorial aggregate, selecting and analyzing only European cities. The European context shows uniform levels of economic development and quality of life and consistent administrative and regulatory systems. This alignment of elements makes statistical comparisons between European cities more proper. Additionally, since the IMD report is based on subjective perceptions, limiting it to a more culturally consistent area avoids biases arising from different social values, political priorities, or urban governance models. The final database therefore includes 56 European smart cities, analyzed according to 39 indicators divided into the technological and infrastructural dimensions (see section “Methodology” for a more detailed description). The dataset includes 39 variables, all of which are based on citizens’ perspectives. The aforementioned indicators can be further classified into five main themes such as health and safety, mobility, activities, opportunities (employment and education), and governance. Indicators can also be grouped under the following two macro-categories: “structure” (infrastructural) and “technology” (technological). This categorization was done in accordance with the conceptual framework used by the Smart City Index. A detailed list of indicators included in both macro-categories is provided in Appendix A to ensure reproducibility.

The initial data corresponding to each city is expressed as a score index calculated by IMD Smart City. It assesses the residents’ perceptions on the technologies and infrastructures available to them in their city. The final score for each city is computed by using the weighted perceptions of the last three years of the survey^45^.

### Methodology

This paper employs a quantitative approach based on the construction of the Pena Distance (DP2) index. The DP2 method is a non-compensatory parametric approach for constructing aggregative indexes. It assigns weights to partial indicators based on their correlation with a global index, capturing the distinctive contribution of each indicator^46–49^. The main advantages of the DP2 method are based on its ability to consider the particular and independent contribution of each indicator, avoiding both the phenomenon of redundancy and heterogeneity^50^. DP2 has been largely applied for the evaluation of economic, social, and environmental indicators^51–54^, demonstrating its effectiveness in dealing with the methodological difficulties that can be found in the multidimensional evaluation, for example, for the analysis of smart cities^10,55,56^.

The calculation of the DP2 method starts with a matrix, V, with the following dimension (k, m), where each element $\:{v}_{kj}$ represents the value of indicator * k* for unit * j*. In the case of the evaluation of the development of smart sustainable cities, the indicators are all positive, and any variation, both positive and negative, regarding the value of the indicators means an improvement or worsening, respectively, for the development of the smart sustainable cities.

Therefore, higher DP2 values indicate a superior citizen-perceived structural and technological performance of smart sustainable cities, as ${d_{kj}}$ represents a greater distance from a theoretical worst-case scenario^54^. The global index $DF\left( j \right)$ is calculated as^50^:

$$DF\left( j \right)=\mathop \sum \limits_{{k=1}}^{K} \frac{{{d_{kj}}}}{{{\sigma _k}}}$$

Here, ${\sigma _k}$ represents the standard deviation of indicator *k*, ensuring standardization. The distance between two statistical units, ${d_{kj}}$, for each partial indicator, is scaled by the inverse of ${\sigma _k}$. In this way, the contribution of each ${d_{kj}}$ to the global indicator is inversely proportional to the standard deviation of its corresponding indicator, and distances with higher variability and vice versa result in less significance.

In the subsequent stage, to avoid redundancy, $DF\left( j \right)$ is adjusted by a correction factor that accounts for linear dependence among indicators. This yields the final DP2 index^49,57^:

$$DP2\left( j \right)=\mathop \sum \limits_{{k=1}}^{K} \frac{{{d_{kj}}}}{{{\sigma _k}}}\left( {1 - R_{{k,k - 1, \ldots ,1}}^{2}} \right)$$

The correction term $R_{{k,k - 1, \ldots ,1}}^{2}$ is the determination coefficient derived from regressing indicator *k* on the preceding indicators. By removing the shared variance, each indicator contributes to the overall DP2 index by providing only its unique information.

DP2 also normalizes the indicators by their standard deviations. This ensures comparability across heterogeneous units of measurement. The method sequentially incorporates indicators into the index, with the first contributing its full variance, while subsequent indicators contribute only their uncorrelated portions. This stepwise process ensures the robustness of the final index by addressing the problems of redundancy and scaling.

The construction of the DP2 index that has been proposed has followed a hierarchical structure with three levels of aggregation. This hierarchical structure is aimed at ensuring conceptual coherence between the theoretical framework and the empirical implementation and maintaining a distinction between infrastructural and technological components:

- First-level aggregation: The individual indicators on aspects of health and safety, mobility, activities, opportunities, and governance are aggregated to obtain their corresponding first-order dimensions, separately for the structural and technological dimensions.
- Second-level aggregation: The five domains are aggregated into two macro-dimensions: structure (infrastructural indicators) and technology (digital/technological indicators).
- Third-level aggregation: This aggregation involves the combination of the secondary factors of infrastructure and technology into a single index called “Overall DP2 Index,” which gives an overall idea about the smart and sustainable performance of the cities in consideration.

All indicators used in the analysis are provided such that higher values correspond to better perceived performance. This is consistent with the semantic structure of the survey items, which ensures a coherent orientation across indicators. As a result, no re-scaling or inversion procedures have been applied to the indicators prior to the application of the DP2 method. In addition, the dataset does not contain missing values for the selected indicators and cities. Therefore, no imputation or data treatment procedures were required.

In this analysis, the ordering of variables follows the theoretical structure reflected by the thematic organization based on conceptual relevance of the Smart City Index. Indicators are incorporated based on the original cluster classification and order within each dimension. In addition, the DP2 corrective system (coefficient of determination) compensates for any redundancies that may occur.

For evaluating the efficiency of 56 smart sustainable cities, the four-quadrant matrix visualization technique is applied. This approach combines the two basic dimensions of structures (X axis) and technology (Y axis). Based on the analysis of these axes, it becomes possible to evaluate the strong and weak aspects of the cities. The size of markers, which correlates with the sum of the DP2 index, helps in determining the level of efficiency of each city. The classification of smart cities into four groups is based on the median values of the structure and technology dimensions, which define the cut-off thresholds for the quadrants. Using the median as the coordinate origin allows for a relative assessment of city performance. Compared to the mean, the median reduces the influence of extreme values, ensures a more balanced partition of the sample, and provides a fair reference for evaluating relative strengths and weaknesses.

The matrix makes it easy to categorize the cities into four different quadrants, each of which represents a particular paradigm of a smart city based on the relative scores of the structure and the technological dimensions:

- top-right quadrant (Group 1) shows cities high-performing in both dimensions.
- top-left (Group 2) and bottom-right (Group 4) quadrants include cities demonstrating strength in one dimension but requiring improvement in the other.
- bottom-left quadrant (Group 3) gathers cities with room for improvement in both dimensions.

This categorization facilitates comparison among cities and synthesizes multi-dimensional data into a clear, interpretable format, helping identify different relative perceived performance of smart cities and areas for improvement. This grouping results from a rule-based classification derived from relative positions within the two-dimensional space.

A robustness assessment was conducted to verify the stability of the results under different grouping conditions. To do so, the analysis was replicated by applying a different threshold to define the quadrant classification. Specifically, median-based and mean-based cut-off values were compared to assess the sensitivity of the resulting groupings. Only 5 out of 56 statistical units changed group assignment when using the mean-based threshold, while the overall pattern of results remained substantively unchanged. This limited variation indicates that the classification is not driven by extreme values and remains stable under alternative specifications. These findings reinforce the descriptive validity of the proposed typology and suggest that the classification is not substantially affected by threshold specification.

## Results

This section provides the results of the comparative analysis of citizen-perceived smart city performance based on DP2 index derived from survey data. The analysis is grounded in subjective evaluations provided by residents, capturing how structural and technological aspects of urban systems are experienced and evaluated. All results presented in this section refer to citizen-perceived performance rather than objective measures of smart city capacity. Table 1 reports the DP2 scores for the first-order dimensions across both structural and technological components, as well as the aggregated results for the second-order dimensions (structure and technology). The overall DP2 index reflects the third-order aggregation of these two macro-dimensions into a single composite measure. It should also be noted that zero values reported in Table 1 represent observed responses in the dataset and do not correspond to missing data.

Considering the structural dimension, the city with the highest perceived structural performance is Zurich, which scores the highest overall in structure (10.63), with relatively high values in the first-order dimensions of perceived health and safety (12.78), opportunities (11.58), and governance (7.21). Oslo (9.24), Copenhagen (9.20), Lausanne (9.03), and Geneve (8.83) follow in the ranking’s first positions. All these cities show high scores in the citizens’ perceptions of the different first-order dimensions, especially in the governance, where they perform in the top five. Rome (0.91) and Athens (0.96) have the lowest scores in the structure dimension. These results are associated with lower scores in governance and opportunities, suggesting comparatively weaker perceived performance in these areas. Nicosia (1.13) shows comparatively low values in mobility and governance from the citizens’ subjective perspective. Bucharest (1.21) and Zagreb (2.23) consistently ranked below the median in all first-order indicators analyzed.

The analysis of the technology dimension shows that Warsaw has the highest perceived technological performance (15.08), with high perceived performance in health and safety (15.81) and governance (12.34). More in general, it proves to be one of the top performers in all the first-order subjective dimensions considered. Vilnius (15.07) and Tallinn (14.72) also report high technological scores, with high investments in smart solutions for health and safety, activities, and opportunities. Among the top five performers based on their rankings are also Bilbao (13.97) and Krakow (13.97), whose performance surpasses the median performance level for all first order dimensions. In contrast to this, Rome (3.27) and Nicosia (3.81) exhibit the highest levels of deficiency in their performance level in terms of technological aspects of performance. Additionally, Hanover (5.13) exhibits weaknesses in relation to its variety of activities and opportunities, whereas Zagreb (6.15) and Marseille (6.88) make up the bottom five performers, underperforming relative to the median in various dimensions of citizens’ perceptions.

With regard to the overall DP2 index that considers citizens’ perceptions in both the technological and structural dimensions, Zurich (6.88) emerges as the city with the highest score in this category, reflecting better perception levels regarding both categories. Following this is Bilbao (6.35) whose level of performance is quite balanced in terms of the structural and technological categories; its rankings in the two categories are about the same. Finally, the top five cities in this dimension include Oslo (6.17), Tallinn (6.16), and Warsaw (6.04). On the other end, poor perceived performers in this case are Rome (0.00), Nicosia (0.25), Bucharest (1.17), Athens (1.18), and Zagreb (1.38).

**Table 1:** Rankings of smart cities’ structural, technological, and overall dimensions.

City	Structure						Technology						Overall
	Health and safety	Mobility	Activities	Opportunities	Governance	Overall structure	Health and safety	Mobility	Activities	Opportunities	Governance	Overall technology	Overall
Amsterdam	6.77	4.51	4.27	8.72	4.74	6.75	11.93	11.31	7.91	9.52	9.76	11.46	4.89
Athens	3.89	1.01	1.29	1.66	0.58	0.96	6.62	7.43	11.62	5.25	5.92	7.56	1.18
Barcelona	6.14	4.25	4.60	8.25	2.35	5.04	9.59	11.13	10.85	9.78	8.09	10.93	3.97
Belfast	6.40	3.23	4.48	9.30	2.70	5.16	9.41	7.56	4.98	11.59	4.77	8.72	3.42
Berlin	7.43	4.33	4.21	6.63	3.49	5.71	7.85	10.04	1.40	5.73	7.50	7.07	3.23
Bilbao	10.14	6.64	6.69	10.60	4.30	8.44	12.64	12.91	15.57	11.08	9.74	13.97	6.35
Birmingham	6.47	3.13	4.20	8.19	4.14	5.87	11.50	9.47	4.60	9.99	8.41	10.09	4.12
Bologna	12.11	4.27	5.83	7.48	3.78	7.17	14.56	11.25	17.11	8.14	9.36	13.68	5.69
Bordeaux	6.07	3.83	6.86	8.80	3.95	6.67	12.00	10.99	7.28	9.65	8.01	10.98	4.73
Bratislava	6.14	2.83	2.01	7.79	3.12	4.42	9.64	7.79	10.47	7.24	5.51	8.91	3.14
Brussels	6.52	3.90	4.69	7.47	3.16	5.48	11.23	10.28	4.47	8.53	9.03	9.91	3.89
Bucharest	2.66	1.20	2.04	4.92	0.23	1.21	9.26	7.04	6.13	6.12	3.64	7.11	1.17
Budapest	3.46	3.03	3.13	6.45	2.19	3.58	7.67	10.16	12.77	9.29	7.96	10.25	3.11
Cardiff	5.80	2.70	5.05	8.89	3.42	5.51	9.93	6.69	9.19	9.34	6.31	9.20	3.71
Copenhagen	11.10	5.11	6.36	11.39	5.81	9.20	11.53	8.17	10.21	10.46	7.66	10.86	5.86
Dublin	3.71	2.31	3.38	8.13	2.63	4.00	5.67	7.11	11.23	8.37	5.74	7.87	2.66
Düsseldorf	9.53	4.40	4.96	8.09	4.57	7.16	9.85	10.82	6.51	7.68	8.47	9.63	4.59
Geneva	9.89	4.73	6.44	9.26	6.05	8.83	11.84	8.67	9.57	8.09	7.82	10.37	5.55
Glasgow	6.53	3.38	5.07	8.12	3.15	5.52	10.59	6.85	3.32	9.02	5.82	8.15	3.44
Gothenburg	7.97	3.95	6.18	9.50	4.16	7.04	9.99	9.23	8.55	8.50	6.26	9.48	4.49
Hamburg	8.87	4.32	5.62	8.80	4.76	7.43	9.30	9.77	5.36	6.44	7.03	8.36	4.37
Hanover	9.28	5.28	5.43	7.93	4.07	7.12	5.46	8.87	0.00	4.93	5.62	5.13	3.36
Helsinki	10.27	6.54	5.84	10.69	5.17	8.78	12.84	9.66	3.83	10.94	8.96	10.81	5.65
Krakow	6.96	4.03	5.04	9.63	4.77	7.00	15.57	11.44	10.85	10.95	11.25	13.97	5.68
Lausanne	10.67	4.80	6.84	10.58	5.73	9.03	9.78	9.46	9.96	9.59	8.65	10.48	5.68
Leeds	7.45	3.05	4.08	8.79	4.12	6.03	12.55	8.02	1.66	10.05	9.56	9.77	4.11
Lille	7.55	4.35	5.37	8.10	4.16	6.68	14.80	12.53	5.74	10.41	9.71	12.55	5.15
Lisbon	5.56	1.87	5.04	4.58	1.63	3.54	10.84	7.79	16.60	11.36	8.37	12.13	3.59
Ljubljana	7.32	2.79	5.04	7.24	2.29	4.82	8.26	10.09	7.02	8.65	4.65	8.53	3.22
London	5.89	3.88	5.47	8.56	3.80	6.15	13.56	12.10	10.21	13.07	10.19	13.67	5.21
Luxembourg	10.28	4.63	6.37	10.30	4.21	7.77	9.83	6.26	11.87	9.26	7.40	9.77	4.91
Lyon	5.98	4.11	6.51	8.36	3.75	6.44	13.33	11.73	11.87	9.70	9.07	12.72	5.09
Madrid	6.45	3.93	5.94	8.68	2.88	5.79	13.71	14.02	10.09	11.53	9.86	13.69	5.05
Manchester	8.03	3.73	4.41	9.26	4.21	6.51	11.15	7.53	4.85	10.72	7.51	9.58	4.27
Marseille	4.05	3.02	3.26	5.06	2.38	3.65	9.54	8.40	2.04	5.52	5.15	6.88	2.23
Milan	9.48	3.24	4.47	5.23	2.26	4.84	13.05	10.97	14.30	5.93	8.11	11.74	4.09
Munich	9.47	4.49	6.11	9.04	5.08	7.94	10.39	10.77	11.62	7.97	8.30	10.83	5.27
Newcastle	9.34	4.65	5.38	9.29	3.93	7.04	11.77	7.10	3.96	9.64	6.97	9.12	4.39
Nicosia	6.51	0.03	1.00	4.26	0.21	1.13	2.42	2.23	6.77	8.91	0.24	3.81	0.25
Oslo	11.64	5.83	6.48	10.97	5.49	9.24	12.19	11.72	10.98	10.41	6.93	11.94	6.17
Paris	4.27	3.09	5.22	6.45	3.36	5.06	13.67	10.81	8.81	7.37	8.16	11.24	4.06
Prague	9.30	4.12	3.95	10.33	4.36	6.91	14.81	9.00	12.64	9.63	8.78	12.65	5.29
Reykjavík	8.22	2.12	4.94	11.23	3.61	6.13	9.90	1.47	16.09	7.74	8.91	9.33	4.03
Riga	6.75	3.65	3.43	6.53	0.96	3.54	10.61	8.11	4.60	7.92	3.30	7.94	2.47
Rome	3.49	0.27	3.32	0.09	0.33	0.91	3.62	5.33	6.51	0.07	3.79	3.27	0.00
Rotterdam	7.52	4.89	4.02	8.60	4.39	6.65	11.99	8.31	3.06	8.66	9.27	9.50	4.32
Sofia	4.44	2.77	2.21	5.68	1.10	2.60	10.58	8.38	5.62	9.33	5.56	8.99	2.32
Stockholm	7.92	3.81	5.80	9.37	4.64	7.20	11.51	8.66	9.06	8.40	7.07	10.09	4.73
Tallinn	11.86	4.88	6.45	9.53	3.60	7.60	14.45	7.65	18.00	12.83	12.21	14.72	6.16
The Hague	8.75	4.52	4.67	8.87	4.65	7.14	10.70	8.18	2.30	8.43	8.12	8.60	4.30
Vienna	10.96	5.54	5.71	9.82	3.92	7.67	10.38	9.88	10.60	10.11	7.82	10.88	5.16
Vilnius	9.12	3.13	4.75	7.05	2.17	5.00	17.48	10.40	13.91	13.62	8.12	15.07	5.06
Warsaw	7.18	4.39	4.78	9.82	4.81	7.11	15.81	12.66	10.72	13.09	12.34	15.08	6.04
Zagreb	3.68	1.18	3.05	6.16	0.89	2.23	5.31	3.82	8.81	8.20	4.23	6.15	1.38
Zaragoza	9.59	5.69	6.28	10.19	3.71	7.55	10.29	10.77	16.34	9.98	8.07	12.19	5.46
Zurich	12.78	5.98	6.34	11.58	7.21	10.63	11.05	11.24	10.47	12.79	8.32	12.22	6.88

**Fig. 1:**
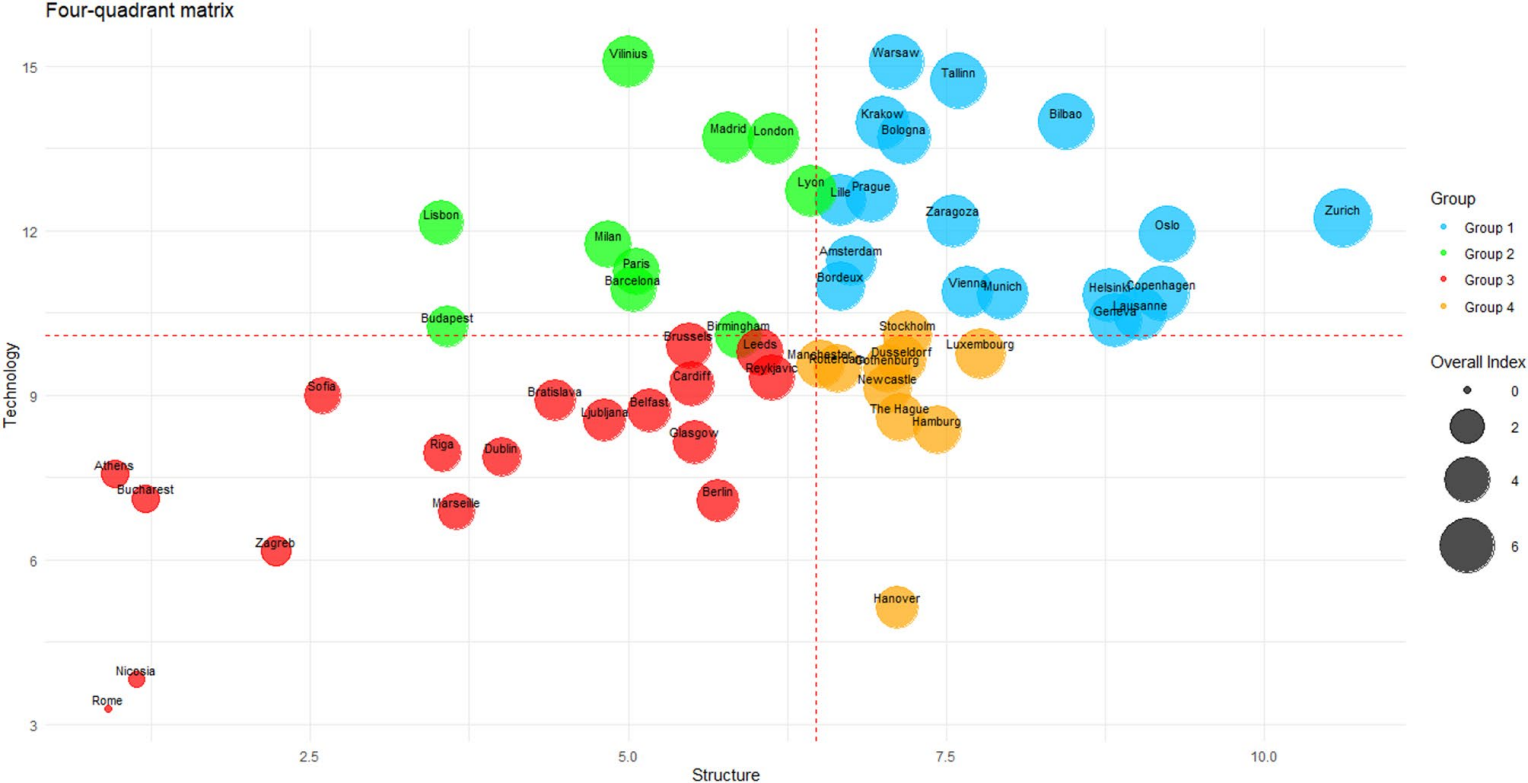
Four-quadrant matrix of smart cities’ paradigms. Source: own elaboration.

The four-quadrant matrix shown in Fig. 1 depicts the 56 smart cities across the two key dimensions of structure (X-axis) and technology (Y-axis). The axes on the graph represent the composite performance of structural and technological factors using the DP2 methodology index. Marker size on the graph is relative to the aggregate DP2 index score, signifying the combination of the two indices into one composite index.

Those cities that lie in the upper right-hand corner (Group 1) have above-median scores in both indices, suggesting a highly-balanced perceived performance in structural and technological factors. Some well-performing cities in Group 1 include Zurich, Bilbao, Oslo, Tallinn, and Warsaw, all represented by large marker sizes, and consequently better DP2 index scores. Meanwhile, cities found in the upper left-hand corner (Group 2) possess above-median scores in technology but below-median in structure. Residents living in Group 2 cities perceive their urban environment as technology-based with relatively weak infrastructure support. Cities such as Vilnius, Madrid, London, and Milan place themselves in this quadrant, focusing on technological development of their urban system. Group 3, in the bottom left quadrant, comprises cities such as Rome, Nicosia, Zagreb, Bucharest, and Athens. These cities are perceived relatively weak in terms of functional integration between structures and technologies. The sizes of the markers are smaller in this group because these cities have lower DP2 index values. Bottom-right quadrant includes Group 4, such as Stockholm, Luxembourg, Düsseldorf, Gothenburg and Manchester, characterized by strong infrastructure but moderate technological integration based on citizens’ judgments.

**Fig. 2:**
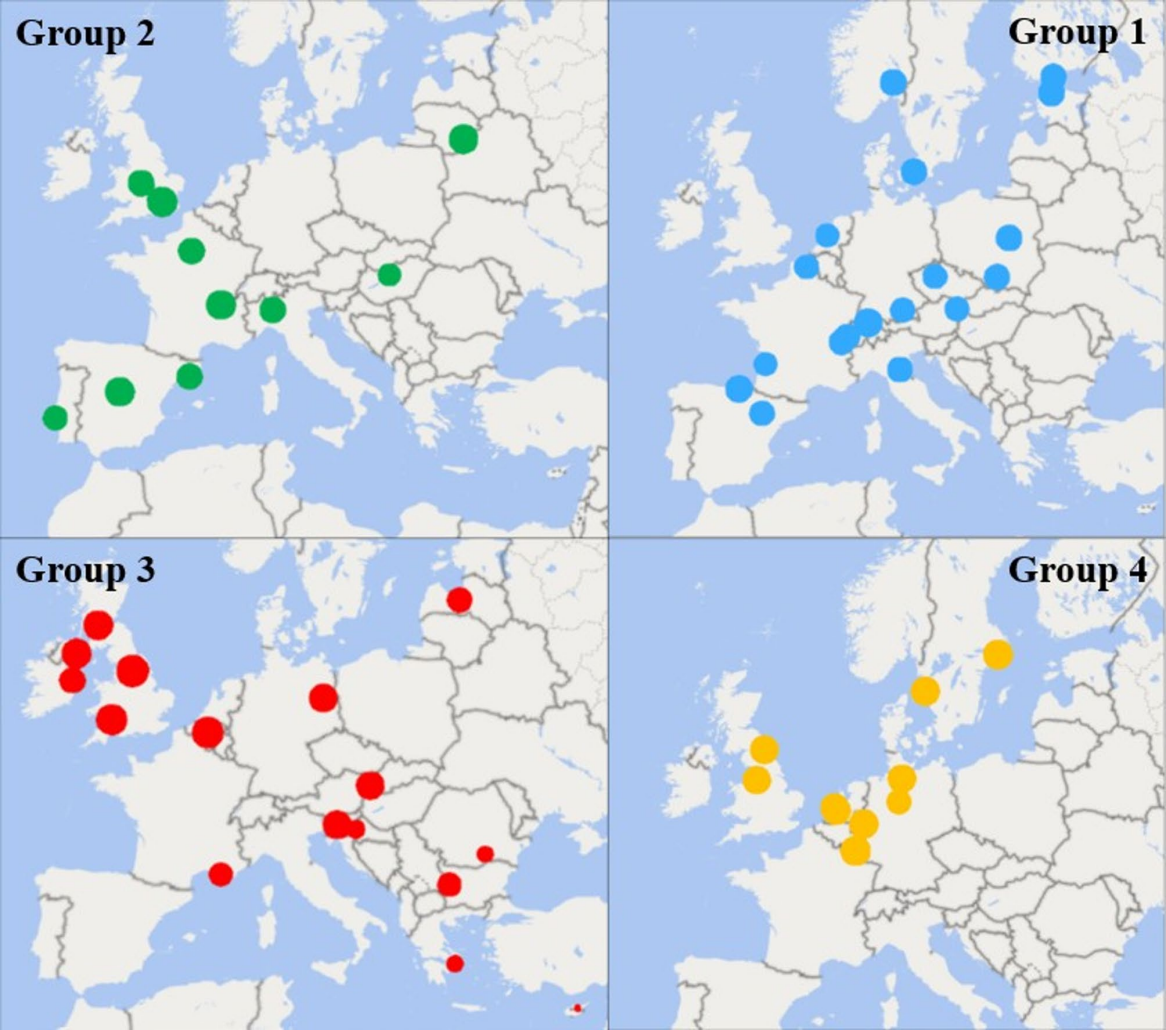
Geographic distribution of cities by four-quadrant group. Source: The cartographic visualization was generated using the “3D Maps” feature of https://www.microsoft.com/it-it/microsoft-365/excel?utm_source=chatgpt.com for Microsoft 365 MSO (Version 2604, Build 16.0.19929.20172, 32-bit).

Based on the previous aggregate results, geographical trends can be identified concerning the performance of smart cities. Figure 2 represents the level of development achieved by smart cities belonging to the four identified groups as a combination of the technology and structure dimensions.

## Discussion

This study provides a comparative and cross-sectional analysis of 56 European smart cities (SC) by examining two key dimensions of sustainability^8,9^. To achieve this, data from the Smart City Index 2023 report, edited by the IMD International Institute for Management Development of SUTD (Singapore University of Technology and Design), was used. These data capture citizens’ perceptions of the main sustainability dimensions of their place of residence. This citizen-centric research is relevant for decision-makers, as it allows them to understand the awareness and satisfaction of citizens with respect to the actions taken by political administrations to make their cities smarter and more sustainable. We have chosen to analyze the data with respect to the Distance-Pena (DP2) method, which is a non-compensatory approach to building aggregate indices, especially necessary for evaluating and comparing complex phenomena, such as the sustainability and smartness of cities. The findings provide a typology of smart city performance based on citizen perceptions. These results should be interpreted as cross-sectional descriptive evidence and do not imply causal relationships or temporal dynamics between groups. In this perspective, they complement—rather than replace—traditional indicators based on objective data, offering additional insight into how residents perceive and evaluate the functioning of their cities.

The results enabled us to categorize the cities into four groups according to their performance, as indicated by DP2 values. As shown in Fig. 1, SCs in the top-right quadrant (Group 1) excel in infrastructure and technology (e.g. Zurich, Oslo and Bilbao); conversely, SCs in Group 3 (bottom-left) struggle in both areas. SCs in Groups 2 and 4 (top-left and bottom-right) display above-median perceived performance in one dimension and below-median perceived performance in the other. These configurations should be interpreted as descriptive patterns observed at a specific point in time. The relative positioning of cities in Fig. 1’s matrix suggests potential complementarities or imbalances between structural and technological dimensions.

**Fig. 3:**
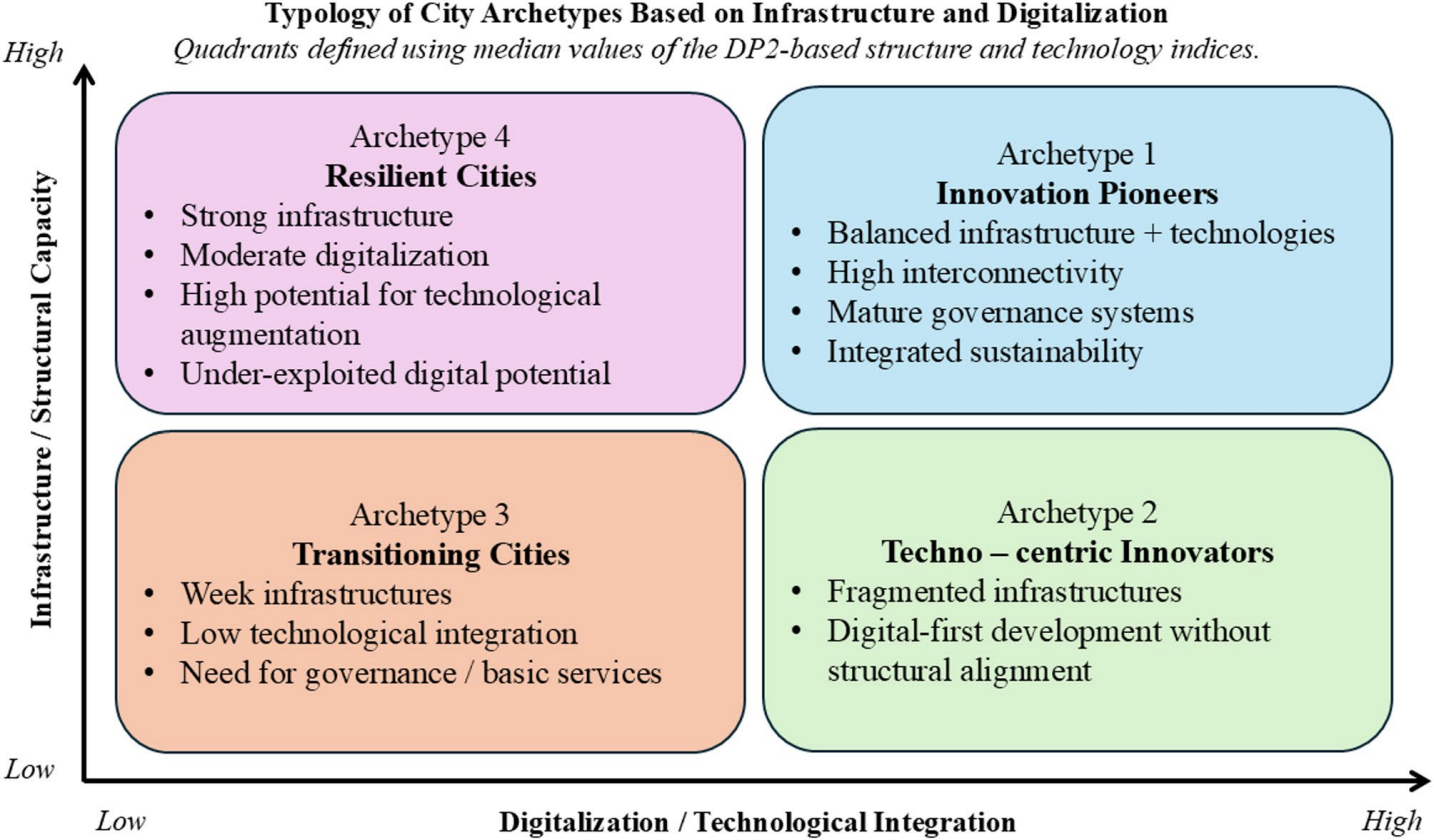
Conceptual summary of the four citizen-perceived smart city archetypes. Source: own elaboration.

From an interpretative perspective, the differences across groups underscore the multidimensional nature of smart city performance and suggest that structural and technological components may be perceived unevenly by residents. In some cases, higher perceived technological performance coexists with weaker evaluations of infrastructure, while in others the opposite pattern emerges. This highlights the importance of jointly considering both dimensions when assessing urban systems. Accordingly, these patterns should be interpreted as relative configurations rather than as indicators of actual development stages. Figure 3 presents a summary of the empirical grouping results within a conceptual two-dimensional space. The typology does not imply a developmental trajectory.

It is important to note the main factors which would enable cities to improve their standing in the eyes of residents. Cities in Group 3 should enhance their infrastructure with investments in mobility (e.g. roads, local public transport, accessible and inclusive stops, lighting), governance, and basic services (e.g. healthcare, education). Cities in Group 4 can improve their position in the citizens’ perceptions by investing in digitalization and developing their technological offerings and cybersecurity. Cities in Group 2 should develop the synergy between infrastructure and digital components.

The key theory underlying our work is based on literature emphasising the importance of using cross-sectional analysis to better interpret data and identify patterns and relationships between variables. This approach is particularly well-suited to identifying the prevalence of certain characteristics across very different groups at a specific point in time^58^. Wang and Cheng^68^ defined them as “*observational studies that analyze data from a population at a single point in time*.” Using a cross-national framework to assess how smart city sustainability indicators influence quality of life (measured by life expectancy) in 25 EU countries, Chovancová et al.,^59^ argue that traditional unified data analysis approaches generate overly general results that fail to capture the specificities and heterogeneity of the individual variables analysed. The purpose of Tang et al.,^60^ is to perform a cross-sectional comparative analysis of sixty global strategic plans for smart cities and to identify ‘archetypes’ based on which types of projects are carried out simultaneously. Thus, the authors emphasize the importance of considering the process of developing a smart city in terms of its non-uniformity. Therefore, the need to evaluate the efficiency of the urban development using a cross-sectional approach emerges.

The methodological originality of our work lies in our analysis of citizen-based data. This is particularly evident when considering the groups to which some cities belong, as determined by our analysis, compared with other studies in literature. For example, the city of Berlin falls into Group 3 in our study, evident difficulties being present in both infrastructural and technological dimensions. However, according to Csukás and Szabó’s^61^ study, which was based on objective data, Berlin is one of the best European smart cities in terms of the variety of “value propositions”. This difference indicates the existence of some possible gap between perception and measurement. This is because, when it comes to citizen perception, some elements of smart city living experience might be overlooked in the objective criteria. As such, the research emphasizes the inadequacy of conventional ranking techniques and the fact that there is an enormous gap between citizen perception and the actual objective data collection. This makes it very important for policy makers to adopt an approach that combines performance-related and citizen-oriented aspects of SCs.

From a practical perspective, this work may provide useful insights to guide strategic urban development decisions. In particular, the findings can support context-specific strategies aimed at improving the balance between structural and technological dimensions based on residents’ experience and perceptions. In addition to planning development strategies for the two dimensions, local government institutions need to monitor the perceptions of the people and establish the priority agenda based on the perceptions, as well as building trust with the institutions. In particular, the subjective data from the people can be used effectively as a complement to the dashboards with the objective parameters. Matching subjective data with objective parameters allows policymakers to determine where to focus public policy efforts, whether on the actual improvement of systems or on marketing and strategic communication.

## Conclusion

This research makes a unique contribution to smart city literature by providing a comparative and citizen-oriented investigation of 56 European urban areas based on perception-based data, drawing on the Smart City Index^9^ and applying the non-compensatory Distance-Pena technique^10,50^. The analysis reveals four groups of smart cities based on their relative position in terms of citizen-perceived structural and technological performance. The findings offer a descriptive typology showing how different dimensions of smart city performance are experienced by residents. Importantly, this typology reflects perceived performance at a specific point in time and should not be interpreted as evidence of developmental trajectories. Cities with above-median values show full integration between the infrastructural and technological dimensions, optimal condition for sustainable and efficient urban systems. On the contrary, other city groups reveal asymmetries across key urban dimensions. These findings should be interpreted as a cross-sectional representation of perceived urban performance rather than as evidence of developmental stages or temporal trajectories. Theoretically, the study highlights the need to integrate technological and human driven approaches^5,6^ and in this way moves the field away from a techno-centric vision towards an inclusive, citizen-based orientation. Firstly, this study shows that urban assessment depends not only on the presence of certain infrastructure and technology in cities, but also on the way in which people perceive and experience such components of smart city development. Methodologically speaking, the DP2 approach demonstrates that it can be successfully applied to analyze a complex and multidimensional phenomenon of smart city performance and provide an objective basis for their assessment. The non-compensatory nature of the DP2 model makes it suitable to use the perception data because it does not allow hiding any deficiencies in specific dimensions.

Despite the above contributions, the study also faces several limitations. Firstly, the fact that data used for analysis was perception-based means that it might be influenced by personal expectations, cultural peculiarities, and the level of knowledge about what actually happens. Therefore, there is a possibility that perception-based information does not completely coincide with objective urban performance. Secondly, the cross-sectional design does not allow conducting any temporal analysis of transitions between different dimensions and understanding any causation between them. Thirdly, the regional scope of research is narrow since only European cities are analyzed; however, such approach ensures better comparability between them. Finally, although the DP2 method reduces the risk of redundancy, results obtained depend on the way in which variables are selected and arranged.

Future research could address these limitations by integrating subjective and objective indicators, expanding the geographical scope of analysis, and adopting longitudinal designs to explore how perceived and measured performance evolve over time. Further studies could also investigate the drivers of perceived performance and examine the relationship between policy interventions and changes in citizen evaluations.

## Data Availability

All data are public and published by IMD Smart City Index 2023. Readers can access them on [https://smacite.eu/en/dissemination/posts/93-imd-smart-city-index-2023](https:/smacite.eu/en/dissemination/posts/93-imd-smart-city-index-2023) .

## Appendix A

List of indicators used in the DP2 index.

**Table:**

Macro-dimension	First-order dimension	Indicator code	Indicator description
Structure	Health & Safety	Health & Safety_S_1	Basic sanitation meets the needs of the poorest areas
Structure	Health & Safety	Health & Safety_S_2	Recycling services are satisfactory
Structure	Health & Safety	Health & Safety_S_3	Public safety is not a problem
Structure	Health & Safety	Health & Safety_S_4	Air pollution is not a problem
Structure	Health & Safety	Health & Safety_S_5	Medical services provision is satisfactory
Structure	Health & Safety	Health & Safety_S_6	Finding housing with rent equal to 30% or less of a monthly salary is not a problem
Structure	Mobility	Mobility_S_1	Traffic congestion is not a problem
Structure	Mobility	Mobility_S_2	Public transport is satisfactory
Structure	Activities	Activities_S_1	Green spaces are satisfactory
Structure	Activities	Activities_S_2	Cultural activities (shows, bars, and museums) are satisfactory
Structure	Opportunities	Opportunities_S_1	Employment finding services are readily available
Structure	Opportunities	Opportunities_S_2	Most children have access to a good school
Structure	Opportunities	Opportunities_S_3	Lifelong learning opportunities are provided by local institutions
Structure	Opportunities	Opportunities_S_4	Businesses are creating new jobs
Structure	Opportunities	Opportunities_S_5	Minorities feel welcome
Structure	Governance	Governance_S_1	Information on local government decisions are easily accessible
Structure	Governance	Governance_S_2	Corruption of city officials is not an issue of concern
Structure	Governance	Governance_S_3	Residents contribute to decision making of local government
Structure	Governance	Governance_S_4	Residents provide feedback on local government projects
Technology	Health & Safety	Health & Safety_T_1	Online reporting of city maintenance problems provides a speedy solution
Technology	Health & Safety	Health & Safety_T_2	A website or App allows residents to easily give away unwanted items
Technology	Health & Safety	Health & Safety_T_3	Free public wifi has improved access to city services
Technology	Health & Safety	Health & Safety_T_4	CCTV cameras has made residents feel safer
Technology	Health & Safety	Health & Safety_T_5	A website or App allows residents to effectively monitor air pollution
Technology	Health & Safety	Health & Safety_T_6	Arranging medical appointments online has improved access
Technology	Mobility	Mobility_T_1	Car-sharing Apps have reduced congestion
Technology	Mobility	Mobility_T_2	Apps that direct you to an available parking space have reduced journey time
Technology	Mobility	Mobility_T_3	Bicycle hiring has reduced congestion
Technology	Mobility	Mobility_T_4	Online scheduling and ticket sales has made public transport easier to use
Technology	Mobility	Mobility_T_5	The city provides information on traffic congestion through mobile phones
Technology	Activities	Activities_T_1	Online purchasing of tickets to shows and museums has made it easier to attend
Technology	Opportunities	Opportunities_T_1	Online access to job listings has made it easier to find work
Technology	Opportunities	Opportunities_T_2	IT skills are taught well in schools
Technology	Opportunities	Opportunities_T_3	Online services provided by the city has made it easier to start a new business
Technology	Opportunities	Opportunities_T_4	The current internet speed and reliability meet connectivity needs
Technology	Governance	Governance_T_1	Online public access to city finances has reduced corruption
Technology	Governance	Governance_T_2	Online voting has increased participation
Technology	Governance	Governance_T_3	An online platform where residents can propose ideas has improved city life
Technology	Governance	Governance_T_4	Processing Identification Documents online has reduced waiting times
